# Viscosities and Densities of Binary and Ternary Mixtures of Aliphatic and Polyaromatic Hydrocarbons: Pyrene +1-Methylnaphthalene + Dodecane at *T* = (293.15 to 343.15) K. Experiment and Modeling

**DOI:** 10.1021/acs.jcim.3c01737

**Published:** 2024-03-19

**Authors:** Maria José Tenorio, Miguel A. Gonzalez, Julia D. Magdaleno, Inmaculada Suárez, Baudilio Coto

**Affiliations:** Chemical, Energy and Mechanical Technology Department, ESCET. Universidad Rey Juan Carlos, c/Tulipán s/n, 28933 Móstoles, Madrid, Spain

## Abstract

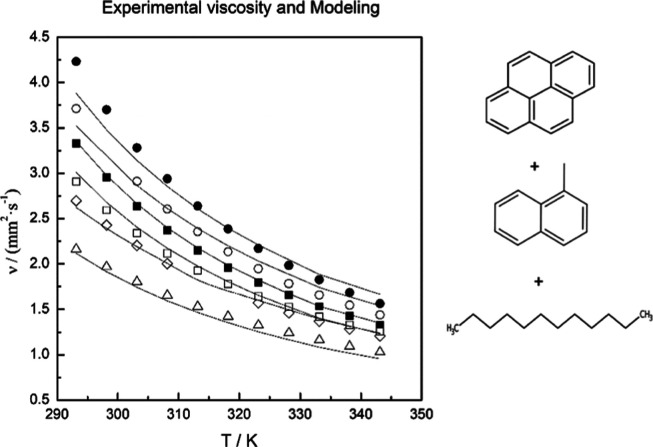

This work presents new experimental viscosity and density data for aromatic and polyaromatic compounds in binary and ternary pyrene, 1-methylnaphthalene, and dodecane mixtures. The lack of experimental viscosity data for these mixtures requires the development of a new database, which is vital for understanding the behavior of mixtures in more complex systems, such as asphaltenes and fuels. The mixtures proposed in this work have been measured over a temperature range of (293.15 to 343.15) K at atmospheric pressure. Several mixture compositions have been studied at these conditions: 1.0, 2.5, 5.0, 7.5, 10.0, 12.5, and 15.0% pyrene mass fraction. The concentration of pyrene correlates with an increase in the viscosity and density values. At the lowest temperature in binary mixtures, the corresponding values reach 4.4217 mPa·s for viscosity and 1.0447 × 10^3^ kg·m^–3^ for density, respectively. In ternary mixtures, the introduction of dodecane leads to the lowest maximum values of 3.5555 mPa·s for viscosity and 1.0112 × 10^3^ kg·m^–3^ for density at the same temperature. The experimental data have been employed for the specific modification of viscosity models. These modifications could facilitate the prediction of the viscosity of mixtures that are more complex than those presented in this work. Various viscosity models have been employed, such as Linear, Ratcliff and Khan, modified UNIFAC-Visco, and Krieger–Dougherty. The settings in the models used reliably reproduce the experiment reliably. However, the Ratcliff model agrees excellently with the experiment, having a low standard deviation (2.0%) compared to other models. Furthermore, a model based on the equation of state of Guo is proposed to predict the viscosity values by modifying the specific parameters and adjusting them to the mixtures proposed in this work. The results from this study are compared to previous work, where pyrene, toluene, and heptane mixtures were analyzed. In this case, we find that the decrease of aggregation grade in the present systems is predicted by the model fixed in this work.

## 1 Introduction

Aviation fuels are highly complex mixtures composed of hundreds of compounds, including alkanes, cycloalkanes, aromatic, and polyaromatic hydrocarbons, and vary depending on the crude oil from which the fuel was derived and in which the refining process was used.^1^ Identifying their composition is challenging, and directly determining their thermophysical properties, such as molecular weight, viscosity, density, surface tension, and refractive index, is even more difficult.^2^ Among these properties, viscosity and density at various temperatures allow the evaluation of new additives for aviation fuels, serving as crucial parameters for economic, ecological, and safety considerations. Viscosity, in particular, is one of the most important physical properties since it is a fundamental parameter in the design and optimization of fuels, biodiesel, jet engines, and injectors.^3,4^ The high viscosity of some fuel mixtures typically results from the presence of solid particles. These particles form emulsions related to heavy components, such as asphaltenes,^5^ whose presence generates significant problems in the oil industry.^6^ The sizes of these particles depend on the pressure, temperature, and composition. Moreover, due to the diverse molecular structures of asphaltenes, aggregates can form due to the π–π interactions between these components, forming large particles with high stability. Analyzing these mixtures experimentally is complex, and to simplify the study, hydrocarbon blends containing a few compounds with similar physical and chemical kinetic properties can be prepared. While there is still a limited scope of experimental studies on the properties of aromatic hydrocarbons and their mixtures,^7,8^ recent publications have explored the combination of experimental and theoretical approaches,^9^ such as molecular dynamics (MD), to investigate thermophysical properties such as viscosity and density. For instance, studies have been conducted on mixtures of cyclohexanes and alkyl cyclohexanes with *n*-paraffins^10−12^ and alkylbenzenes with linear alkanes.^13,14^

The viscosities of complex mixtures can be estimated by empirical correlations, semitheoretical, and theoretical models.^15^ Empirical methods use^6^ information on mixture density and operating conditions, but poor results are obtained and have no predictive capacity 6; in most cases, they are only of application for very specific systems.^4^

Semitheoretical models include parameters that must be determined from experimental information. The Roelands model contains two parameters to evaluate the viscosity variation of mineral oils with temperature.^16^ Group contribution (GC) methods require a binary interaction parameter matrix.^17^ More theoretical methods include quantitative structure–property relationships, equations of state (EoS),^15^ and molecular dynamics.^10,11,13^

Recently, methods based on EoS have received high attention. Guo et al. (2001)^18^ used the Peng–Robinson equation’s formulation and transformed the variables T–V–P to P–η–T. The model introduced accurate T and P dependence for the viscosity of light compounds, but it failed for heavier ones. Several new versions have been proposed, improved, and extended.^15,19,20^ The objective is to maintain the simple equation form, to describe simultaneously the liquid and gas phases, and to improve the extension of the model to heavier components.

Mixing rules describe the composition effect and allow for extension to mixtures. Reference (21) differentiates the pure mixing rules, including the linear model and those based on the viscosity blending index,^22^ and mixing rules with additional binary parameters, including those with a named excess function.^23^ Chevalier et al.^17^ predicted the mixture’s kinematic viscosity from the pure components’ values and deviations determined by a GC method, the UNIFAC-Visco model. Methods based on EoS have the advantage of straightforwardly applying well-known mixing rules of the cubic equations of state to the formulation with viscosity.^15,19,20^

In a different approach, the viscosity models of a colloidal suspension use volume fractions and parameters that describe interactions between particles and the continuous phase. For example, Krieger and Dougherty’s model^24^ determines the relative viscosity as a function of solvent viscosity and intrinsic viscosity.

This study presents new experimental data on the dynamic viscosity and density of aromatic compounds in binary and ternary mixtures formed by pyrene, 1-methylnaphthalene, and dodecane covering from (293.15 to 343.15) K at atmospheric pressure and several compositions: 1.0, 2.5, 5.0, 7.5, 10.0, 12.5, and 15.0% mass fraction of pyrene. Since experimental viscosity values for these mixtures are practically nonexistent, this is an important database that can be used to develop theoretical viscosity models to predict the viscosity of complex mixtures. Several kinds of viscosity models were considered: linear,^21^ Ratcliff and Khan,^25^ modified UNIFAC-Visco,^26^ and Krieger–Dougherty^27^ to compare the results with those published in previous work for pyrene, toluene, and heptane mixtures.^26^ In addition, an EoS proposed by Guo et al.^18^ was analyzed to predict viscosity values, obtaining specific parameters for pure components that form the mixtures of pyrene, 1-methylnaphthalene, and dodecane.

## 2 Experimental Section

### 2.1 Materials

Chemicals used in this work are listed in Table 1 next to CAS N°, supplier, and mass fraction purity. The supplier specified purities, and all chemicals were used without further purifications.

**Table 1:** Chemicals Used in This Work

name	CASR no.	formula	supplier	mass fraction purity
dodecane	112-40-3	C_12_H_26_	Acros Organics	99%
1-methylnaphthalene	90-12-0	C_11_H_10_	Acros Organics	96%
pyrene	129-00-0	C_16_H_10_	Alfa Aesar	99%

### 2.2 Sample Preparation and Viscosity and Density Measurements

The binary and ternary mixtures were prepared at the same composition range of polyaromatic compound: 1.0, 2.5, 5.0, 7.5, 10.0, 12.5, and 15.0 wt % of pyrene, and for the ternary mixtures for each of these pyrene compositions, the mass ratios of solvents methylnaphthalene/dodecane, *r* (g/g), were: 0.1, 0.5, 1.0, 5.0, and 10.0. Mixtures were prepared following the procedure previously described.^26,28^ Briefly, pyrene and 1-methylnaphthalene were weighed at room temperature and atmospheric pressure using a Sartorius balance (model: 15205974) with an accuracy of ±0.0001 g and added to a glass 100 mL vial with a Teflon magnetic bar. The mixtures were kept at a constant temperature (313.15 K) and stirred in a silicon bath to ensure a homogeneous mixture. A corresponding amount of dodecane was added to prepare the ternary mixtures. These mixtures were stirred at a constant temperature (323.15 K) for 12 h. Those mixtures with a higher concentration of pyrene and dodecane could not be measured because the pyrene did not completely dissolve. The standard deviation of the composition for the samples was 0.1%.

Dynamic viscosity and density measurements were carried out using the Stabinger SVM 3001/G2^29^ at several temperatures. The temperature was determined with an integrated Pt100 sensor. It was controlled to ±0.01 K. First, the sample was heated at 323.15 K in a silicon bath to completely dissolve the possible solid phase. The sample was injected using a syringe at 323.15 K, and measurements were made in a cooling ramp from 343.15 to 293.15 K at a 5 K interval.

The expanded uncertainties were calculated considering the deviations in the measurements based on the accuracy of the instrument and the measurement method.^30^ Standard uncertainties providing a level of confidence of approximately 95% for density is 1 × 10^–1^ kg·m^–3^ and for dynamic viscosity is 7.0 × 10^–4^ mPa·s. In addition, a standard type APS3 (Lot. No.: 3200405) supplied by the manufacturer (Anton Parr) was chosen to measure its density and viscosity from 293.15 to 343.15 K. The data obtained in this work were compared to certificate of calibration, and the expanded uncertainties reported were estimated at 1 × 10^–1^ kg·m^–3^ for density and 4.0 × 10^–4^ mPa·s for dynamic viscosity.

## 3 Calculation Section

Well-known viscosity models describe the effects of temperature^31,32^ and composition.^21^

Table 2 lists all of the viscosity models used in this work, including the modifications introduced to improve the accuracy of the description of the experimental behavior. The Roelands model^33^ was used to describe pyrene viscosity as a function of temperature. The Ratcliff and Khan model^25^ calculates mixture viscosity from pure component values and a function, Δ(ln ν), representing the deviation from linearity. According to previous results,^26^ the deviation function is given by Δ(ln ν)_t3_ with *a*_*ij*_ temperature independents.

**Table 2:** Viscosity Models^a^

model	equation	parameters	composition	refs
Roelands	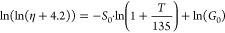	*S*_0_, *G*_0_		(33)
Linear	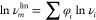		ϕ_*i*_	
Ratcliff	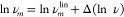	*a*_*ij*_	*w*_*i*_	(25)
	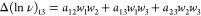			
UNIFAC-Visco	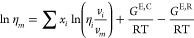	*a*_*nm*_*a*_*i*_	*x*_*i*_*w*_*i*_	(34)
	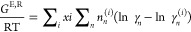			
	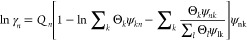			
	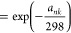			
	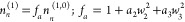			
Krieger	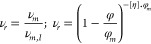	[η]; φ_*m*_	ϕ_*i*_	(27)
	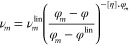			
	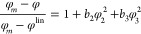	*b*_*i*_		
Guo	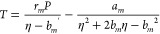	*k*_i_*k*_ij_	x_i_	(18)
	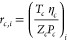			
	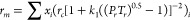			
	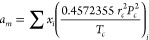			
	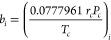			
	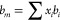			
	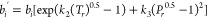			
	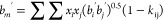			

a η: dynamic viscosity, ν: kinematic viscosity, *T*: temperature, *P*: pressure, *w*: mass fraction, φ: volume fraction, *x*: mole fraction, VBI: viscosity blending index, *v*: molar volume, *G*^E^: excess Gibbs energy, γ: activity coefficient, Θ: group area fraction, Ψ_nm_: interaction parameter-temperature UNIFAC function, *n*_*n*_^(*i*)^: number of *n*-group in the *i*-component, [η]: intrinsic viscosity of the Krieger model, subscript *i* and *j* refer to components *i* and *j*, *m* to the mixture, *n*, *l*, and *k* to the groups, *c* to critical properties, and *r* to reduced properties.

The UNIFAC-Visco model^34^ allows viscosity predictions from the binary interaction parameters between groups *n* and *m*, *a*_*nm*_, included in the interaction parameter-temperature function Ψ_*nm*_. As described,^35^ the value for temperature in Ψ_*nm*_ is replaced by the reference value 298 K, and the temperature effect is introduced through the pure component viscosities and the pure and mixture molar volumes. However, available interaction parameters are scarce.

A possible association process of pyrene can be computed as a change in the number of UNIFAC groups, molecular structural parameters, *r* and *q*, and molecular weight but without effect in the *a*_*nm*_ interaction parameters. An association factor, *f*_a_, was introduced according to *n*_*k*_^(1)^ = *f*_*a*_*n*_*k*_^(1,0)^, where *n*_*k*_^(1,0)^ represents the number of *k*-groups in component 1 (pyrene) as a single molecule, if *f*_*a*_ > 1, the number of groups increases to *n*_*k*_^(1)^, and values for *r*, *q*, and *M*_w_ will be also increased representing that association occurs. The association factor was assumed composition-dependent, by *f*_*a*_ = 1 + *a*_2_*w*_2_^2^ + *a*_3_*w*_3_^2^.

As an example of specific models considering the mixture as a dispersed system, the model of Krieger–Dougherty^27^ was used. Relative viscosity, defined as , where ν_m,l_ is the kinematic viscosity for a liquid in the absence of the dispersed phase, depends on the volume fraction, φ, of the dispersed phase and includes as parameters the maximum volume fraction of the dispersed phase, φ_*m*_, and the intrinsic viscosity, [η].^36^

Combining the definition of linear viscosity and the Krieger model, and assuming that nonlinearity affect only φ but not φ_*m*_ and [η], the following expression can be obtained:  when the ratio  is higher than 1 due to φ < φ^lin^, a disaggregation process occurs. In this work, it was assumed that pyrene is responsible for such a change. Such a ratio was correlated against composition, .

Guo model^18^ is described in Table 2 in its standard form. To reduce the description in Table 2, a combination of pure and mixture properties is presented. From the well-known Peng–Robinson equation, T–V–P is replaced by P–η–T in, R by *r*, and *a* and *b* are used with similar meanings. New magnitude *b*′ replaces the covolume in the first term; it is computed from *b* and includes the dependence of P and T. A key property is the critical viscosity, η_c_, difficult to obtain experimentally and unavailable in most cases. In this work, it was computed using the correlation described in ref (37). Pure component properties are described by obtaining specific *k*_1_, *k*_2_, and *k*_3_ values, which introduced the temperature and pressure dependence of *r* and *b*′. The *k*’s values are commonly correlated to additional pure component parameters (acentric factor, critical properties, and number of carbons), and several versions deal with such correlations.^18,19^ Mixing rules are the classical ones with binary parameters in the *b*_m_′ property.

## 4 Results and Discussion

### 4.1 Experimental Data

**Figure 1:**
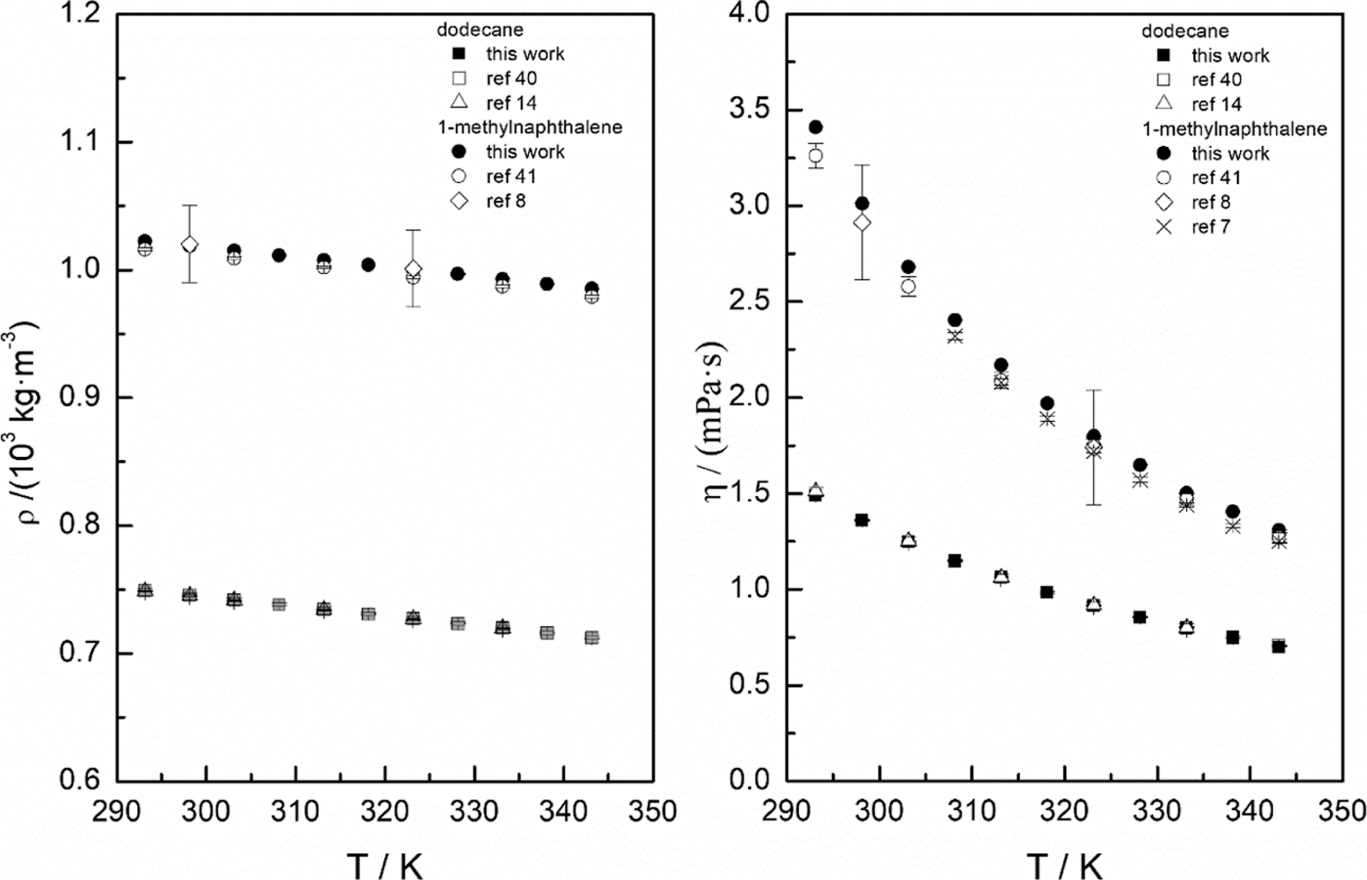
Comparison between experimental dynamic viscosity and density data of dodecane and 1-methylnaphthalene measured in this work and literature data from Table 3.

Experimental data on the density and dynamic viscosity of pure 1-methylnaphthalene and dodecane and binary and ternary systems are given in Tables 3–5, respectively. All values were obtained at a temperature range from 293.15 to 343.15 K at a 5 K interval. Due to pyrene being solid at this temperature range, as described in a previous publication, dynamic viscosities of pure pyrene were extrapolated by the Roelands model, and considering a linear density–temperature relationship, the density values of pure pyrene were calculated using data obtained from Hind et al.^38,39^ The dynamic viscosity and the density values of pure liquid compounds obtained in this work have been compared with the data obtained in the literature (Table 3 and Figure 1).^7,8,14,40,41^Figure 1 plots all data listed in Table 3, experimental dynamic viscosity and density values of this work, and literature data. Deviations obtained from the uncertainties reported in the literature and in this work have been incorporated as error bars into the plot. As shown in the figure, density values show minor variations, while disparities between measured and reported viscosity values are more significant. Nevertheless, the measured data from this study fall within the deviations of the data set. Despite impurities being responsible for significant errors in measurements of densities and viscosities,^42^ the good agreement found in this work for pure component values and those of the bibliography allows us to discard such error sources. Experimental and computed data for pure 1-methylnaphthalene, dodecane, and pyrene were employed to fit the models outlined in the calculation section.

**Table 3:** Experimental Density (ρ) and Dynamic Viscosity (η) of Pure Liquids at *T* = (293.15 to 343.15) K and Atmospheric Pressure (*P* = 101.6 kPa) and Literature Values^a^

compound	*T*/*K*	ρ/(10^3^ kg·m^–3^)	η/(mPa·s)	ρ/(10^3^ kg·m^–3^) lit.	η/(mPa·s) lit
dodecane	293.15	0.7490	1.4902	0.74944^b^; 0.74879^c^	1.4885^b^; 1.51^c^
	298.15	0.7455	1.3597	0.74573^b^; 0.74516^c^	1.3589^b^
	303.15	0.7418	1.2481	0.74203^b^; 0.74153^c^	1.2462^b^; 1.25^c^
	308.15	0.7382	1.1506	0.73834^b^	1.1477^b^
	313.15	0.7345	1.0641	0.73464^b^; 0.73425^c^	1.0610^b^; 1.06^c^
	318.15	0.7309	0.9855	0.73095^b^	0.98426^b^
	323.15	0.7272	0.9144	0.72726^b^; 0.72694^c^	0.91601^b^; 0.916^c^
	328.15	0.7235	0.8533	0.72356^b^	0.85500^b^
	333.15	0.7199	0.7971	0.71986^b^; 0.71959^c^	0.80023^b^; 0.798^c^
	338.15	0.7161	0.7452	0.71616^b^	0.75083^b^
	343.15	0.7120	0.6998	0.71245^b^	0.70612^b^
1-methylnaphthalene	293.15	1.0225	3.4093	1.016^d^	3.26^d^
	298.15	1.0188	3.0110	1.020^e^	2.913^e^
	303.15	1.0151	2.6811	1.009^d^	2.58^d^
	308.15	1.0113	2.4040		2.32^f^
	313.15	1.0076	2.1701	1.002^d^	2.09^d^; 2.08^f^
	318.15	1.0039	1.9701		1.89^f^
	323.15	1.0001	1.7980	0.994^d^; 1.001^e^	1.75^d^; 1.740^e^; 1.72^f^
	328.15	0.9967	1.6483		1.57^f^
	333.15	0.9929	1.5025	0.987^d^	1.47^d^; 1.44^f^
	338.15	0.9891	1.4054		1.33^f^
	343.15	0.9853	1.3095	0.979^d^	1.27^d^; 1.25^f^

a Standard uncertainties (u) are u(*T*) = 0.01 K, u(*P*) = 0.05 kPa, u(ρ) = 1.0 × 10^–1^ kg·m^–3^, and u(η) = 4.0 × 10^–4^ mPa·s.

b Reference (40).

c Reference (14).

d Reference (41).

e Reference (8).

f Reference (7).

**Table 4:** Experimental Density (ρ) and Dynamic Viscosity (η) of Binary System Pyrene (1) + 1-Methylnaphthalene (2) at *T*= (293.15 to 343.15) K and Atmospheric Pressure (101.6 kPa)^a^

*T*/*K*	ρ/(10^3^ kg·m^–3^)	η/(mPa·s)	ρ/(10^3^ kg·m^–3^)	η/(mPa·s)	ρ/(10^3^ kg·m^–3^)	η/(mPa·s)	ρ/(10^3^ kg·m^–3^)	η/(mPa·s)
	w_1_ = 0.010		w_1_ = 0.025		w_1_ = 0.050		w_1_ = 0.075	
293.15	1.0186	3.3905	1.0212	3.5012	1.0243	3.6439	1.0279	3.8292
298.15	1.0149	2.9946	1.0173	3.1044	1.0206	3.2082	1.0240	3.3616
303.15	1.0112	2.6648	1.0139		1.0169	2.8435	1.0202	2.9712
308.15	1.0073	2.3905	1.0102	2.4734	1.0132	2.5471	1.0166	2.6524
313.15	1.0038	2.1565	1.0066	2.2289	1.0096	2.2686	1.0129	2.3770
318.15	1.0001	1.9585	1.0029	2.0202	1.0059	2.0716	1.0095	2.1553
323.15	0.9964	1.7873	0.9993	1.8358	1.0023		1.0058	1.9518
328.15	0.9927	1.6463	0.9956	1.6853	0.9986		1.0019	1.7883
333.15	0.9890	1.5168	0.9919	1.5603	0.9949	1.6038	0.9983	1.6494
338.15	0.9853	1.4094	0.9882	1.4419	0.9912	1.4754	0.9949	1.5128
343.15	0.9816	1.3039	0.9846		0.9875	1.3708	0.9912	1.4051
	w_1_ = 0.100		w_1_ = 0.125		w_1_ = 0.150			
293.15	1.0360	4.0091	1.0411	4.2191	1.0447	4.4217		
298.15	1.0323	3.5217	1.0372	3.6873	1.0409	3.8523		
303.15	1.0286	3.1099	1.0338	3.2463	1.0373	3.4020		
308.15	1.0250	2.7745	1.0302		1.0336	3.0369		
313.15	1.0213	2.4851	1.0265		1.0300	2.7160		
318.15	1.0176	2.2470	1.0225	2.3555	1.0263	2.4479		
323.15	1.0139	2.0330	1.0192	2.1347	1.0227	2.2186		
328.15	1.0102		1.0155	1.9512	1.0190	2.0203		
333.15	1.0066		1.0117	1.7907	1.0153	1.8524		
338.15	1.0029		1.0080	1.6497	1.0115	1.7043		
343.15	0.9995	1.4544	1.0038	1.5277	1.0072	1.5749		

a Standard uncertainties (u) are u(*T*) = 0.01 K, u(*P*) = 0.05 kPa, u(ρ) = 1.0 × 10^–1^ kg·m^–3^, u(η) = 4.0 × 10^–4^ mPa·s, and u(*w*_1_) = 0.001.

**Table 5:** Experimental Density (ρ) and Dynamic Viscosity (η) of Ternary Mixture: Pyrene (1) in 1-Methylnaphthalene (2) and Dodecane (3) at Different Mass Ratios, *r* (g/g), 1-Methylnaphthalene/Dodecane, at *T*= (293.15 to 343.15) K, and Atmospheric Pressure (101.6 kPa)^a^

*T*/*K*	*w*_1_	ρ/(10^3^ kg·m^–3^)	η/(mPa·s)	ρ/(10^3^ kg·m^–3^)	η/(mPa·s)	ρ/(10^3^ kg·m^–3^)	η/(mPa·s)	ρ/(10^3^ kg·m^–3^)	η/(mPa·s)	ρ/(10^3^ kg·m^–3^)	η/(mPa·s)
		*r* (g/g) = 0.1		*r* (g/g) = 0.5		*r* (g/g) = 1.0		*r* (g/g) = 5.0		*r* (g/g) = 10.0	
293.15	0.010	0.7713	1.5401	0.8265	1.6688	0.8670	1.8763	0.9661	2.6156	0.9945	2.8930
298.15		0.7676	1.4059	0.8228	1.5126	0.8632	1.6996	0.9624	2.3375	0.9908	2.5686
303.15		0.7639	1.2841	0.8191	1.3812	0.8593	1.5489	0.9586	2.1026	0.9870	2.3075
308.15		0.7603	1.1855	0.8153	1.2669	0.8557	1.4176	0.9549	1.9048	0.9833	2.0816
313.15		0.7566	1.0960	0.8116	1.1711	0.8520	1.3055	0.9511	1.7330	0.9795	1.8876
318.15		0.7529	1.0161	0.8079	1.0829	0.8483	1.2068	0.9474	1.5868	0.9757	1.7324
323.15		0.7492	0.9455	0.8041	1.0141	0.8444	1.1205	0.9436	1.4577	0.9719	1.5962
328.15		0.7455	0.8812	0.8004	0.9428	0.8407	1.0430	0.9399	1.3455	0.9683	1.4778
333.15		0.7418	0.8245	0.7966	0.8864	0.8370	0.9728	0.9361	1.2328	0.9645	1.3694
338.15		0.7380	0.7717	0.7928	0.8339	0.8331	0.9101	0.9323	1.1570	0.9606	1.2775
343.15		0.7338	0.7226	0.7883	0.7926	0.8290	0.8534	0.9285	1.0816	0.9562	1.2012
293.15	0.025	0.7756	1.5654	0.8306	1.7120	0.8719	1.9161	0.9696	2.7088	0.9968	2.9759
298.15		0.7719	1.4261	0.8268	1.5522	0.8682	1.7330	0.9659	2.4181	0.9931	2.6386
303.15		0.7682	1.3058	0.8231	1.4166	0.8644	1.5750	0.9622	2.1730	0.9894	2.3609
308.15		0.7645	1.2025	0.8194	1.3023	0.8607	1.4405	0.9584	1.9654	0.9856	2.1279
313.15		0.7609	1.1090	0.8157	1.1955	0.8570	1.3213	0.9547	1.7881	0.9818	1.9329
318.15		0.7572	1.0297	0.8119	1.1083	0.8533	1.2173	0.9509	1.6341	0.9779	1.7664
323.15		0.7535	0.9577	0.8082	1.0303	0.8496	1.1258	0.9472	1.5011	0.9744	1.6154
328.15		0.7498	0.8929	0.8045	0.9631	0.8458	1.0435	0.9434	1.3825	0.9706	1.5101
333.15		0.7461	0.8352	0.8007	0.9042	0.8420	0.9614	0.9397	1.2678	0.9669	1.4017
338.15		0.7423	0.7835	0.7968	0.8501	0.8383	0.9063	0.9359	1.1895	0.9629	1.3054
343.15		0.7381	0.7377	0.7925	0.8024	0.8345	0.8629	0.9320	1.1167	0.9584	1.2291
293.15	0.050	0.7828	1.6149	0.8374	1.7839	0.8780	2.0140	0.9743	2.8499	1.0009	3.1085
298.15		0.7791	1.4723	0.8336	1.6201	0.8743	1.8178	0.9705	2.5393	0.9970	2.7502
303.15		0.7755	1.3415	0.8299	1.4773	0.8706	1.6503	0.9668	2.2780	0.9933	
308.15		0.7718	1.2332	0.8262	1.3534	0.8669	1.5051	0.9631	2.0562	0.9899	
313.15		0.7681	1.1367	0.8225	1.2481	0.8631	1.3790	0.9594	1.8679	0.9860	2.0030
318.15		0.7644	1.0585	0.8188	1.1574	0.8594	1.2693	0.9556	1.7046	0.9823	1.8317
323.15		0.7608	0.9843	0.8150	1.0745	0.8556	1.1734	0.9519	1.5640	0.9785	1.6873
328.15		0.7571	0.9182	0.8113	1.0009	0.8519	1.0866	0.9481	1.4397	0.9748	1.5525
333.15		0.7533	0.8570	0.8075	0.9355	0.8482	0.9993	0.9444	1.3172	0.9710	1.4451
338.15		0.7494	0.8069	0.8037	0.8773	0.8444	0.9394	0.9406	1.2359	0.9671	1.3414
343.15		0.7449	0.7637	0.7995	0.8225	0.8407	0.8802	0.9369	1.1575	0.9625	1.2582
293.15	0.075	0.7900	1.6985	0.8438	1.9026	0.8841	2.1268	0.9787	2.9765	1.0047	3.2614
298.15		0.7864	1.5496	0.8402	1.7143	0.8804	1.9146	0.9750	2.6480	1.0007	2.8862
303.15		0.7828	1.4121	0.8365	1.5594	0.8767	1.7365	0.9714	2.3724	0.9971	2.5708
308.15		0.7791	1.3012	0.8327	1.4265	0.8730	1.5810	0.9677	2.1419	0.9936	
313.15		0.7754	1.1987	0.8290	1.3104	0.8693	1.4465	0.9639	1.9803	0.9901	
318.15		0.7717	1.1129	0.8253	1.2130	0.8655	1.3290	0.9602	1.8034	0.9861	1.9003
323.15		0.7680	1.0331	0.8216	1.1252	0.8619	1.2261	0.9564	1.6493	0.9824	1.7520
328.15		0.7644	0.9605	0.8179	1.0487	0.8581	1.1342	0.9527	1.5126	0.9787	1.6141
333.15		0.7606	0.8977	0.8141	0.9775	0.8544	1.0423	0.9490	1.3762	0.9750	1.4865
338.15		0.7569	0.8429	0.8103	0.9182	0.8508	0.9812	0.9452	1.2851	0.9712	1.3816
343.15		0.7528	0.7949	0.8060	0.8629	0.8470	0.9666	0.9414	1.1954	0.9672	1.2810
293.15	0.100	0.7975	2.1297	0.8508	1.9455	0.8900	2.1463	0.9836	3.1417	1.0088	3.3971
298.15		0.7939	1.9481	0.8471	1.7511	0.8863	1.9326	0.9799	2.7865	1.0053	2.9909
303.15		0.7903	1.8069	0.8433	1.5898	0.8825	1.7506	0.9762	2.4915	1.0017	2.6563
308.15		0.7867	1.6757	0.8396	1.4533	0.8788	1.5934	0.9725	2.2437	0.9980	2.4112
313.15		0.7831	1.2193	0.8359	1.3359	0.8750	1.4572	0.9688	2.0315	0.9941	2.1758
318.15		0.7795	1.1271	0.8322	1.2277	0.8713	1.3375	0.9651	1.8518	0.9902	1.9796
323.15		0.7758	1.0468	0.8285	1.1404	0.8675	1.2331	0.9614	1.6947	0.9867	1.7976
328.15		0.7721	0.9727	0.8248	1.0606	0.8638	1.1409	0.9577	1.5587	0.9828	1.6539
333.15		0.7684	0.9108	0.8210	0.9915	0.8600	1.0460	0.9540	1.4228	0.9792	1.5233
338.15		0.7646	0.8550	0.8172	0.9287	0.8563	0.9845	0.9502	1.3314	0.9753	1.4112
343.15		0.7605	0.8041	0.8131	0.8702	0.8525	0.9230	0.9465	1.2400	0.9709	1.3104
293.15	0.125			0.8584	2.0375	0.8981	2.2941	0.9888	3.211	1.0112	3.5555
298.15				0.8546	1.8326	0.8944	2.0607	0.9851	2.824	1.0075	3.1303
303.15				0.8509	1.6634	0.8906	1.8609	0.9814	2.5232	1.0038	2.7788
308.15				0.8472	1.5178	0.8869	1.6903	0.9777	2.2651	1.0001	2.4847
313.15				0.8435	1.3985	0.8832	1.5451	0.9739	2.0544	0.9964	2.2365
318.15				0.8398	1.2944	0.8795	1.4203	0.9701	1.8855	0.9927	2.0246
323.15				0.8361	1.1993	0.8758	1.3123	0.9665	1.7255	0.9890	1.8466
328.15				0.8324	1.1123	0.8720	1.2175	0.9628	1.5866	0.9853	1.6895
333.15				0.8286	1.0371	0.8683	1.1379	0.9591	1.4642	0.9816	1.5533
338.15				0.8248	0.9712	0.8644	1.0639	0.9553	1.3615	0.9778	1.433
343.15				0.8207	0.9090	0.8599	1.0006	0.9512	1.2646	0.9736	1.3305
293.15	0.150			0.8651	2.134	0.9061	2.4433	0.9976	3.3704	1.0165	3.7736
298.15				0.8614	1.9200	0.9023	2.1895	0.9939	2.9988		
303.15				0.8577	1.7416	0.8986	1.9793	0.9902	2.6656	1.0085	2.9344
308.15				0.8540	1.5858	0.8947	1.7923	0.9865	2.4064	1.0049	2.6178
313.15				0.8503	1.4522	0.8912		0.9828	2.1737	1.0011	2.3575
318.15				0.8466	1.3369	0.8874		0.9791	1.9767	0.9974	2.1290
323.15				0.8429	1.2374	0.8837	1.3894	0.9753	1.8064	0.9938	1.9349
328.15				0.8391	1.1502	0.8800	1.2863	0.9714	1.6564	0.9901	1.7652
333.15				0.8354	1.0712	0.8763	1.1994	0.9678	1.5335	0.9865	1.6364
338.15				0.8316	1.0009	0.8725	1.1211	0.9635	1.4263	0.9828	1.5197
343.15				0.8276	0.93702	0.8682	1.0493	0.9586	1.3331	0.9791	1.4087

a Standard uncertainties (u) are u(*T*) = 0.01 K, u(*P*) = 0.05 kPa, u(ρ) = 1.0 × 10^–1^ kg·m^–3^, u(η) = 4.0 × 10^–4^ mPa·s, u(*w*_1_) = 0.001, and u(*r*) = 0.002.

**Figure 2:**
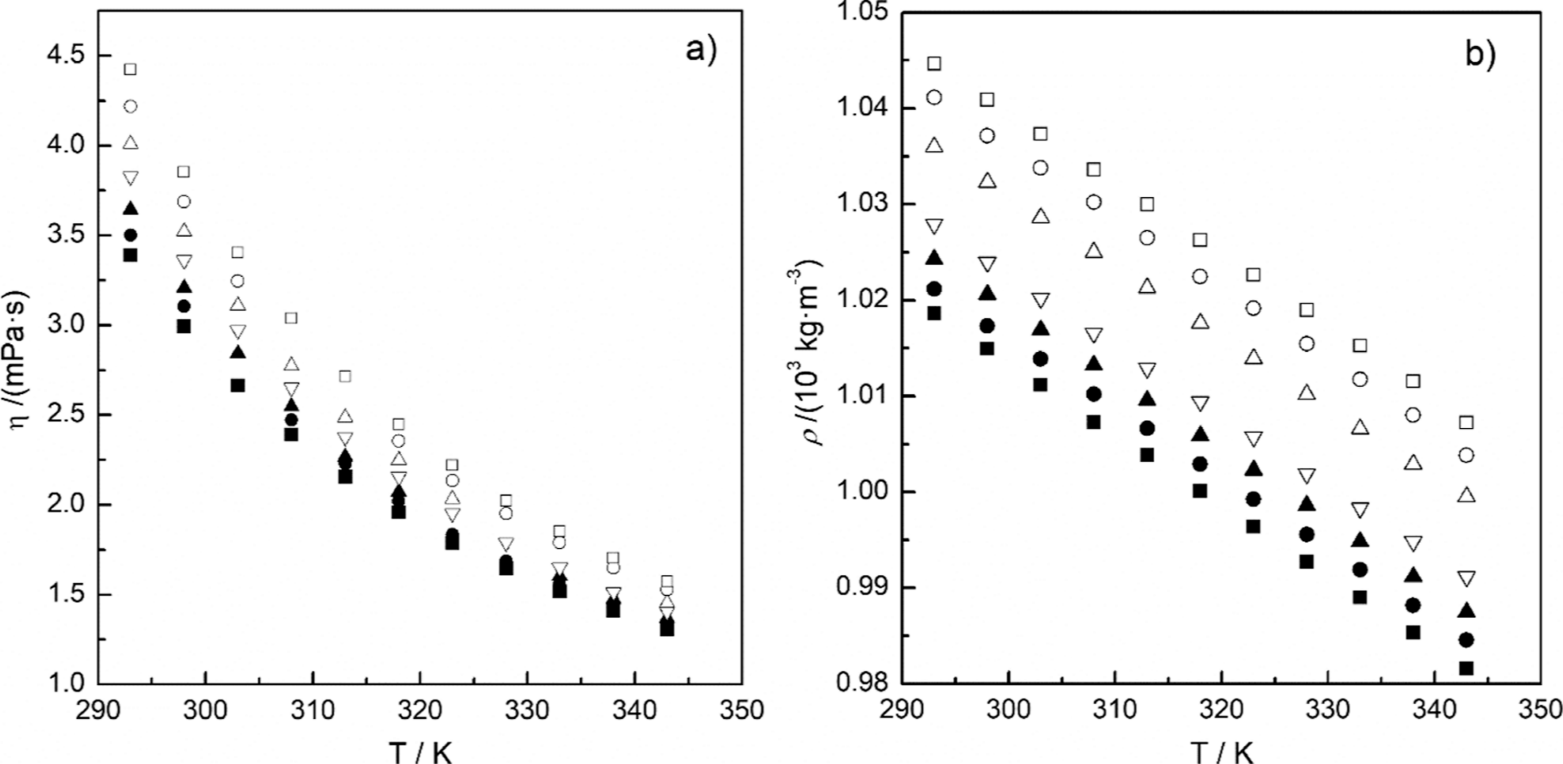
Dynamic viscosity (a) and density (b) as a function of temperature for binary mixtures pyrene (1) + 1-methylnaphthalene (2). (■) 1.0 wt %; (●) 2.5 wt %; (▲) 5.0 wt %; (▽) 7.5 wt %; (Δ) 10.0 wt %; (○) 12.5 wt %; and (□) 15.0 wt % of pyrene.

Table 4 presents the experimental dynamic viscosities and densities for binary mixtures of pyrene and 1-methylnaphthalene. The measurements were performed for different mass fractions of pyrene: 1.0, 2.5, 5.0, 7.5, 10.0, 12.5, and 15.0 mass %. In addition, Figure 2a,b represent these data graphically. As expected, both viscosity and density values increase with the increasing pyrene concentration at a constant temperature. Conversely, the viscosity and density decrease with increasing temperature for each binary mixture with the same composition. In all these systems, pyrene was dissolved at all temperatures and compositions studied, unlike those observed in the previous work,^26^ in which the appearance of a solid phase was observed in 15.0 wt % pyrene in toluene at the lowest temperature. This suggests that pyrene exhibits higher solubility in 1-methylnaphthalene compared to toluene, likely due to a stronger resemblance between the two compounds.

For the ternary systems, pyrene + 1-methylnaphthalene + dodecane, the measured densities and dynamic viscosities from (293.15 to 343.15) K and atmospheric pressure are given in Table 5. Experimental data indicate that the densities and viscosities of all ternary mixtures decrease as the temperature increases. For a given pyrene composition, an increase in the dodecane content in the mixture leads to a decrease in both density and viscosity values. Notably, in two ternary mixtures with high dodecane content, a solid phase emerges, specifically those mixtures with a mass ratio of 1-methylnaphthalene/dodecane 0.1 (g/g) throughout the studied temperature range. This phenomenon was observed only in mixtures containing 12.5 and 15 w % pyrene and compared with the previous work,^26^ in which the appearance of solid was already observed at lower concentrations of pyrene (7.5 w %) in the ternary mixtures with toluene and heptane.

**Figure 3:**
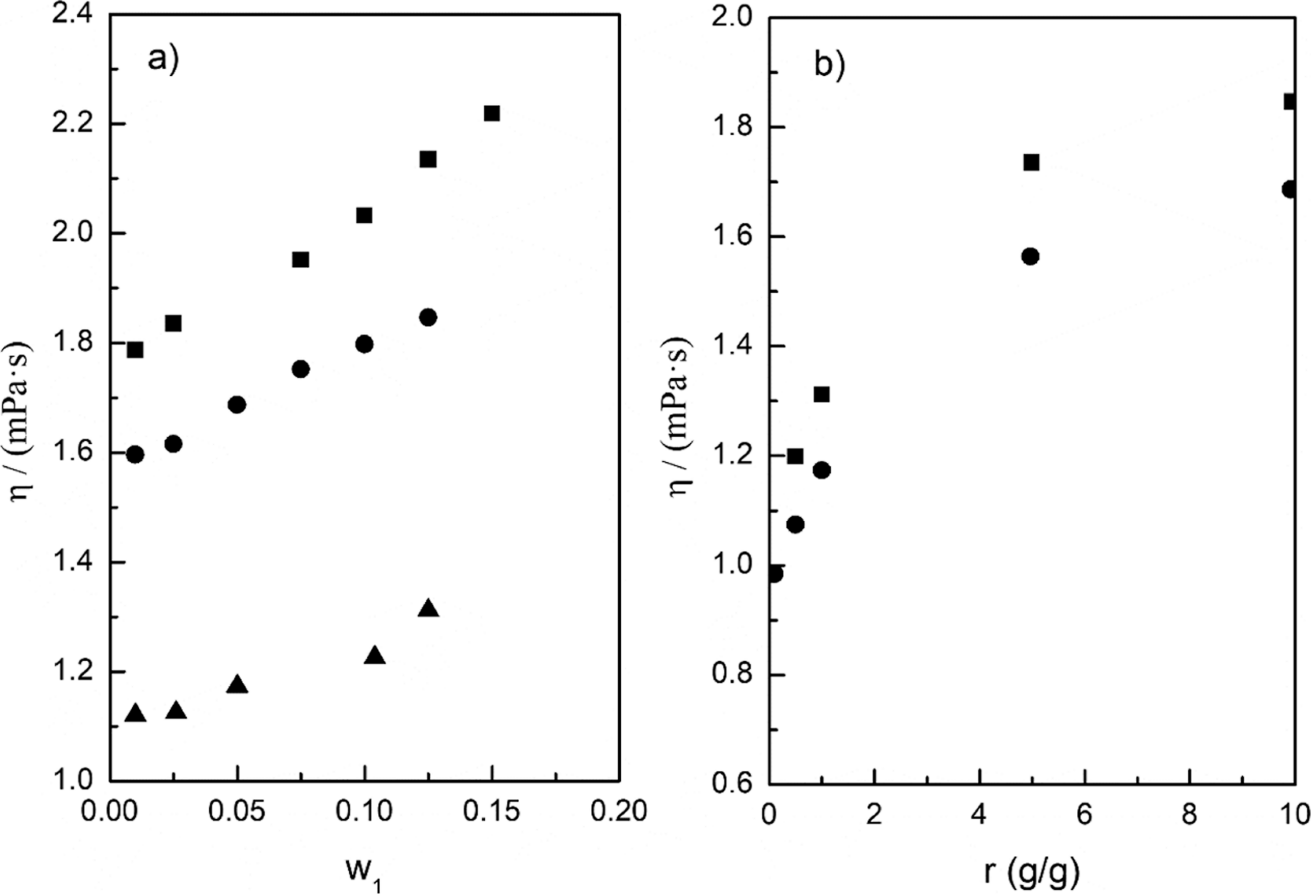
(a) Dynamic viscosity vs *w*_pyrene_ for the binary system: pyrene +1-methylnaphthalene (■), and ternary systems with *r* (g/g) = 10 (●) and *r* (g/g) = 1 (▲). (b) Dynamic viscosity vs *r* for ternary systems with *w*_pyrene_ = 0.125 (■) and *w*_pyrene_ = 0.05 (●).

Figure 3 displays, as an example, some selected data to check the effect of single components. Figure 3a represents dynamic viscosity vs mass fraction of pyrene (*w*_1_) for three systems and clearly shows a linear viscosity increase proportional to the content of pyrene, the compound with the highest viscosity. Such an increase is relatively lower when ratio 1-methylnaphthalene/dodecane decreases as the amount of dodecane is higher, the compound with the lower viscosity. Figure 3b represents dynamic viscosity vs mass ratio for two different pyrene compositions and shows a viscosity increase as ratio is higher due to the reduction of the dodecane component.

Some measurements, in both binary and ternary mixtures, could not be performed due to the mixtures’ low viscosity and density values. The instrument’s rotor was forced to rotate, resulting in unreliable viscosity measurements that lacked sufficient precision.^43^ As a result, these measures were excluded from the data in Tables 4 and 5.

### 4.2 Viscosity Models

The accuracy of viscosity models was checked, and the optimization of parameters was carried out according to the standard deviation and percent relative absolute deviation
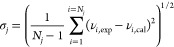
1a
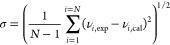
1b
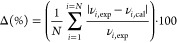
1c
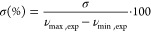
1d
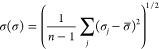
1e

Where subscript exp and cal refer to experimental and calculated values, max and min refer to the highest and lowest viscosity value, and *j* and *i* refer to a system or an individual data; sums on *i* are extended over all the experimental data, *N*_*j*_ for system *j*, and *N* for all the data. In eq 1e, σ̅ is the average of all the σ_*j*_, and the sum on *j* is extended to all the systems, *n*. Table 6 lists the obtained parameters for all of the viscosity models used in this work.

**Table 6:** Viscosity Model Parameters^a^

model	parameters
Roelands (pyrene)	*S*_0_ = 0.729
	*G*_0_ = 4.964
Ratcliff, Δ(ln ν)_t3_	*a*_12_ = 1.042
	*a*_13_ = 0.951
	*a*_23_ = −0.689
UNIFAC-Visco	*a*_2_ = −0.5
	a_3_ = 1.4
Krieger	[η] = 0.151
	φ_m_ = 0.503
	*b*_2_ = 0.396
	*b*_3_ = 0.959
Guo	1: *k*_1_ = 1261; *k*_2_ = −12.83; *k*_3_ = 0.0
	2: *k*_1_ = 2700; *k*_2_ = −15.19; *k*_3_ = 0.0
	3: *k*_1_ = 0.967; *k*_2_ = −14.80; *k*_3_ = 1.43

a Subscript: (1) pyrene, (2) 1-methylnaphthalene, and (3) dodecane.

Table 7 lists the overall values for σ, Δ(%), and σ(%) of each model, and the standard deviation of the deviations, σ(σ). Table S1 lists the numerical values of the standard deviation for each studied system individually, corresponding to each model.

**Table 7:** Overall Standard Deviations of all Models

	Linear	Ratcliff	Krieger	UNI-Visco Table 8	UNI-Visco with f_a_	Guo
σ	0.150	0.066	0.138	0.196	0.179	0.240
σ (%)	4.6	2.0	4.2	6.0	5.5	7.4
Δ (%)	5.3	2.4	4.7	7.2	6.4	11.6
σ (σ)	0.098	0.052	0.090	0.123	0.110	0.098

#### 4.2.1 Linear Model

Overall values listed in Table 7, σ = 0.150 mm^2^·s^–1^, Δ(%) = 5.3, and σ(%) = 4.6, can be regarded as accurate and compare favorably with more complex models. However, when individual systems are analyzed, different accuracies for systems depending on their composition are obtained, as can be checked in Figure S1. The linear model performance is equivalent to previous study,^26^ higher σ, 0.150 mm^2^·s^–1^ vs 0.031 mm^2^·s^–1^, are related with higher viscosity values, and comparison of percent deviation shows similar values, 5.3 vs 5.0 for Δ(%) and 4.6 vs 6.5 for σ(%).

#### 4.2.2 Ratcliff Model

**Figure 4:**
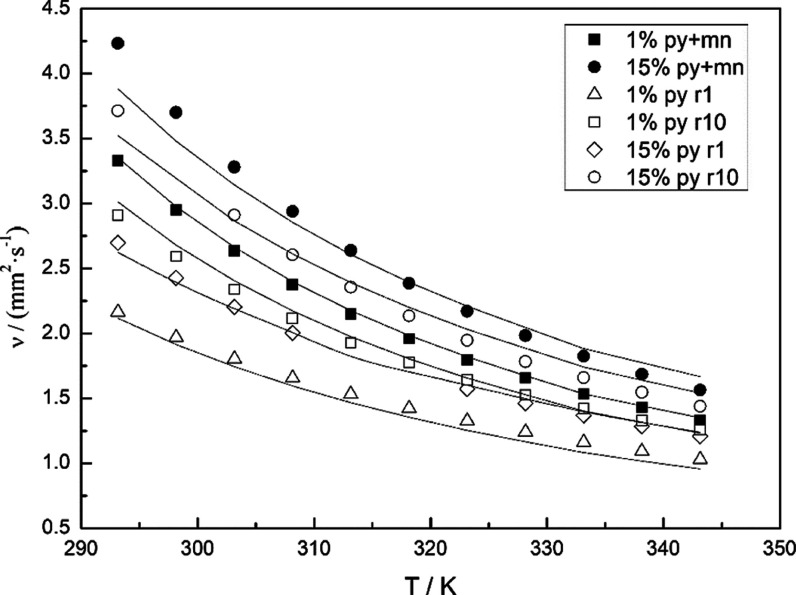
Kinematic viscosity vs temperature of binary systems pyrene + 1-methylnaphthalene: (■) 1.0% pyrene; (⬤) 15% pyrene; and ternary systems pyrene + 1-methylnaphthalene + dodecane with *r* (1-methylnaphthalene/dodecane, g/g) = 1, and *r* = 10: (△) 1.0% pyrene *r* = 1.0; (□) 1.0% pyrene *r* = 10; (◇) 15% pyrene *r* = 1.0; and (◯) 15% pyrene *r* = 10. Solid line: Ratcliff model.

**Figure 5:**
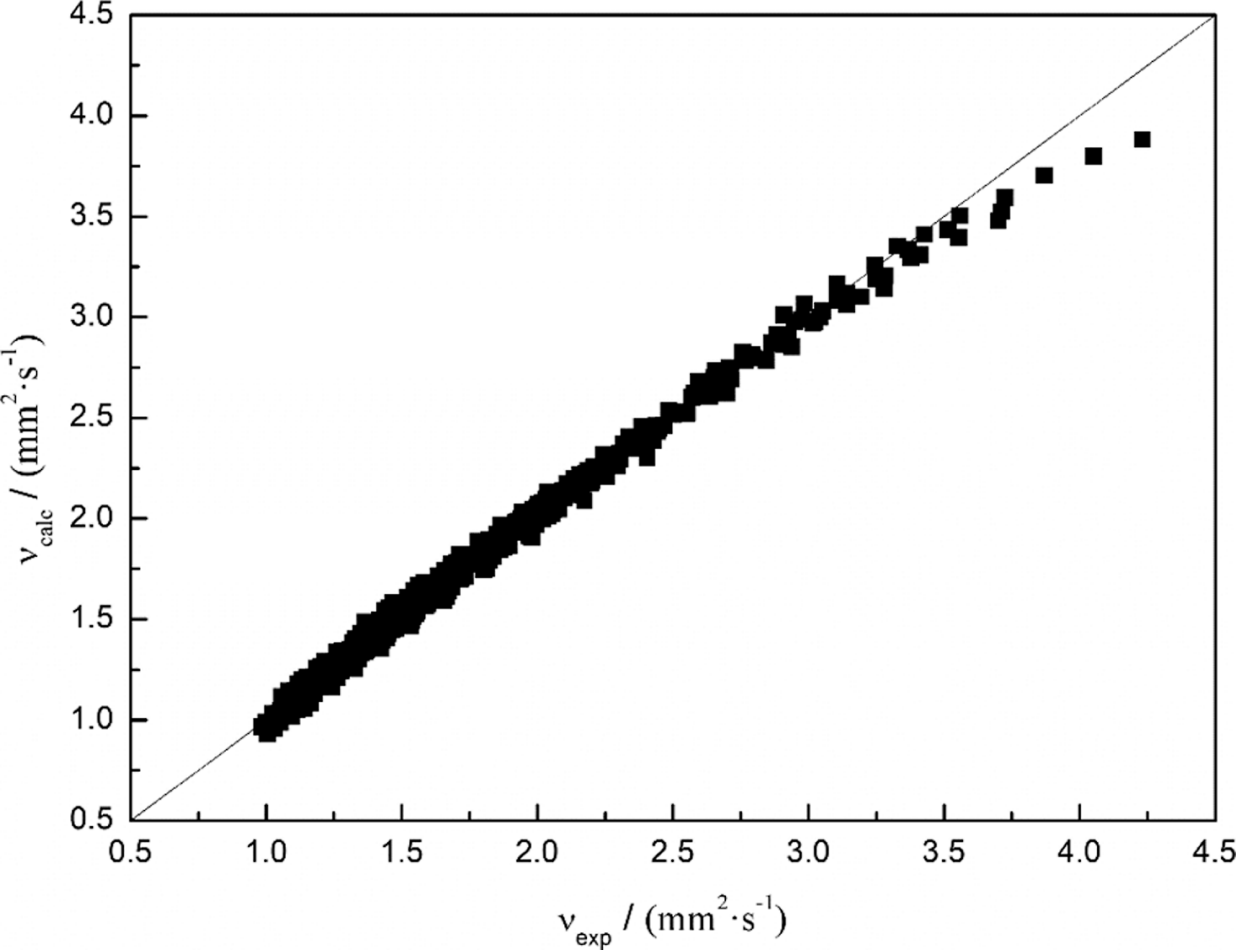
Evaluating the Ratcliff model against experimentally obtained values for kinematic viscosities.

Δ(ln ν)_t3_ requires three parameters, obtained values were *a*_12_ = 1.042, *a*_13_ = 0.951, and *a*_23_ = −0.689. Overall deviation values in Table 7, σ = 0.066 mm^2^·s^–1^, Δ(%) = 2.4, and σ(%) = 2.0, represent an important reduction for the deviations. For example, Figure 4 shows the temperature effect for binary systems with 1 and 15% of pyrene and ternary systems with 1% and 15% of pyrene and *r* (1-methylnaphthalene/dodecane, g/g) equal to 1 and 10. The calculated values by the Radcliff model are included. In addition, Figure 5 plots all the calculated viscosity values (Ratcliff model) vs experimental data to show the agreement. Figure S1 represents deviations for individual systems for the Ratcliff model; the overall performance can be considered to be improved.

Compared to previous work,^26^ similar values of percent deviations, 2.4 vs 2.5 for Δ(%) and 2.0 vs 3.2 for σ(%) were obtained. It is relevant the different values obtained for parameter *a*_12_, equal to 1.042 for pyrene/1-methylnaphthalene and 0.115 for pyrene/toluene, which can be related to the strongest interaction between pyrene/1-methylnaphthalene, probably because of the higher size of the aromatic rings.

#### 4.2.3 Krieger Model

From the fitting process, a value of φ_*m*_ = 0.503 was obtained, in agreement with literature (φ_*m*_ = 0.58).^44^ Intrinsic viscosity has a low value, [η] = 0.151, which can be related to the tiny size of the eventually associated species and the low viscosity of the solvent.^45^ Values for *b*_2_ = 0.396 and *b*_3_ = 0.959 are positive; thus, φ < φ^lin^ and disaggregation occurs, stronger for dodecane than that for 1-methylnaphthalene. Results from Table 7, σ = 0.138 mm^2^·s^–1^, Δ(%) = 4.7, and σ(%) = 4.2, can be considered accurate and represent improvement versus the linear model.

Values of percent deviations are similar to those of previous work;^26^ however, when parameters are compared, intrinsic viscosity has a lower value, 0.151 vs 0.253, representing smaller eventual associated species; *b*_2_ and *b*_3_ are always positive, with higher *b*_2_ values for 1-methylnaphthalene than toluene, representing a high disaggregation.

#### 4.2.4 UNIFAC-Visco Model

According to previous experience,^26^ improved results were obtained with the aromatic group AC (aromatic carbon without hydrogen), but ACH and AC were the same main group (the same interaction parameter), with the same values for R and Q as in the UNIFAC model for activity coefficients, and the following definition was used: pyrene = 12 ACH+6 AC groups, 1-methylnaphthalene = 7 ACH+3 AC + 1 CH_3_ groups. Only one binary interaction was recalculated; in this work, it was assumed that CH_2_-ACH has the most considerable effect and was fitted. Table 8 lists the final UNIFAC-Visco structural and interaction parameters. Deviations in Table 7, σ = 0.196 mm^2^·s^–1^, Δ(%) = 7.2, and σ(%) = 6.0, represent that UNIFAC-Visco is less accurate than the linear viscosity model.

**Table 8:** Structural and Binary Interaction Parameters for the UNIFAC-Visco Model with Fitted Parameters

			*a*_nm_/*K*			
	R	Q	CH_2_	CH_3_	ACH	AC
CH_2_	0.674	0.540	0.00	66.53	23.00	23.00
CH_3_	0.901	0.848	–709.50	0.00	–119.50	–119.50
ACH	0.531	0.400	–21.00	237.20	0.00	0.00
AC	0.365	0.120	–21.00	237.20	0.00	0.00

The method to account for the eventual association of pyrene led to more accurate viscosity predictions, from Table 7, σ = 0.179 mm^2^·s^–1^, Δ(%) = 6.4, and σ(%) = 5.5, but they are still worse than the linear model. The obtained parameters were *a*_2_ = −0.5 and *a*_3_ = 1.4, representing that 1-methylnaphthalene leads to a reduction in association and the opposite for dodecane.

Figure S2 represents deviations for individual systems for UNIFAC-Visco model versions. Predictions are less accurate than those of the linear model due to the systematic deviations found for some specific compositions.

The UNIFAC-Visco model performance is equivalent to previous study,^26^ with similar percent deviation values, 7.2 vs 5.6 for Δ(%) and 6.0 vs 8.1 for σ(%). However, accounting for association effects leads to a significant deviation reduction for systems pyrene + toluene + heptane but only a slight decrease for systems pyrene +1-methylnaphthalene + dodecane. In addition, similar *a*_2_ values are obtained for 1-methylnaphthalene and toluene, but lower *a*_3_ values, 1.4 for dodecane vs 2.3 for heptane. Thus, a decrease in the association process can be considered, in agreement with the analysis of previous models.

#### 4.2.5 Guo Model

Several proposed EoS were checked to predict pure component dynamic viscosities.^18−20^ The Guo model^18^ leads to better results; only their results are presented here.

**Figure 6:**
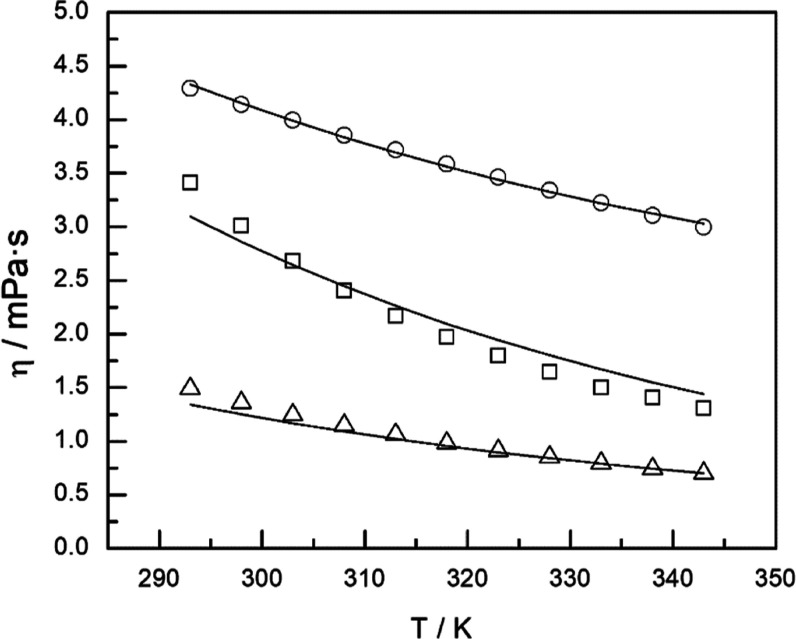
Dynamic viscosity as a function of temperature for pure components: (○) pyrene; (□) 1-methylnaphthalene; (Δ) dodecane; (line) calculated values by Guo’s model.^18^

Two sets of pure component parameters were considered,^18,19^ but only satisfactory results were obtained for dodecane. This can be due to the lack of experimental data for the polyaromatic compounds and the fact that most correlations are obtained exclusively from monoaromatics,^19^ even when heavy compounds are considered. For this reason, new parameter values for pyrene and 1-methylnaphthalene were obtained by fitting the experimental data obtained in this work. The value for *k*_3_ = 0 was set as all the experimental values were at atmospheric pressure. Values for dodecane were received by the correlation reported in ref (18) which yielded the best results. The final parameters for the three components are listed in Table 7, and the calculated values for dynamic viscosity are plotted in Figure 6. Values σ for such calculations are 0.023, 0.161, and 0.069 mPa·s for pyrene, 1-methylnaphthalene, and dodecane, respectively. Such results can be considered very accurate for pyrene [σ(%) = 1.8], but not for 1-methylnaphthalene, and dodecane [σ(%) around 10].

**Figure 7:**
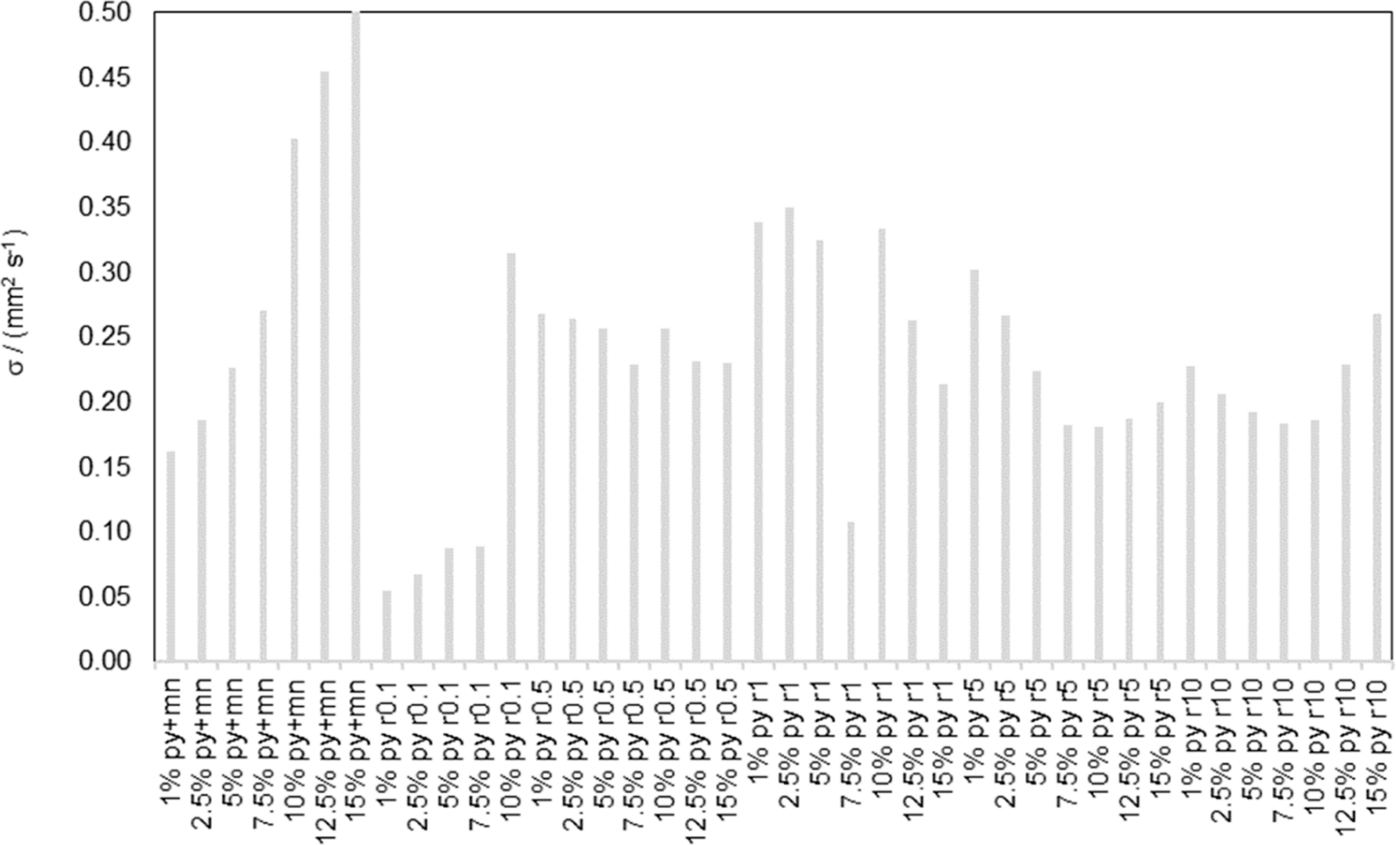
Standard deviations, σ, for all the systems studied using the Guo model.^18^

The application of this model to mixtures was done without fitted binary parameters, to determine the model’s ability to predict the mixtures’ viscosity from the calculated pure component values. Poor descriptions of pure 1-methylnaphthalene and dodecane introduce a limitation that would lead to large deviations for the mixtures. Deviations in Table 7, σ = 0.24 mm^2^·s^–1^, Δ(%) = 11.9, and σ(%) = 7.5, represent values only slightly higher than that for UNIFAC and can be considered reasonable values as this model was used a fully predictive model for mixtures. Figure 7 represents deviations for individual systems for the Guo model. Predictions are less accurate than the rest of the models, as expected from the results for the pure components.

## 5 Conclusions

In the present study, viscosities and densities of pyrene mixtures in binary systems with 1-methylnaphthalene and ternary systems adding *n*-dodecane were investigated in terms of pyrene composition and temperature. To account for these variables, different models have been used, such as the Roelands model, Ratcliff and Khan model, and modified UNIFAC-Visco, Krieger and Guo EoS.

The dynamic viscosity and the density values of pure liquid compounds were measured, and it is possible to check density values show minor variations, in comparison with viscosity values probably due to the influence of the impurities on these measurements. The pyrene concentration influences the binary mixtures, increasing the values of the dynamic viscosities and densities. Experimental data indicate that the densities and viscosities of all ternary mixtures decrease as the temperature increases.

Viscosity models were analyzed by standard deviation and percent relative absolute deviation for these mixtures. Linear model results can be compared favorably with more complex models but with higher σ values which are related to higher viscosity values. The Ratcliff model, which requires three parameters, represents an important reduction for the deviations. From these parameters, it is possible to study the interaction between pyrene and other components of the mixture 1-methylnaphthalene or toluene related to aromaticity and size of these aromatic rings. From the study with the Krieger model, intrinsic viscosity has a low value, which can be related to the tiny size of the eventually associated species and the low viscosity of the solvent; this model slightly improved on the linear model with its standard deviation. The UNIFAC-Visco model method to account for the eventual association of pyrene led to more accurate viscosity predictions, but they are still worse than those of the linear model. Lastly, predictions of Guo’s model presented a standard deviation higher than the rest of the models, as expected from the results of the pure components.

## Data Availability

All data are included in tables and figures provided in the manuscript and Supported Information is available on GitHub https://github.com/mjts2023/Data.git.

## Supporting Information Available

Standard deviations of all mixtures and all models; comparison of standard deviations, σ, for all the systems studied using the linear model, Ratcliff model Δ(ln ν)_t3_, and Krieger model; and comparison of standard deviations, σ, for all the systems studied using the UNIFAC-Visco model: group AC and fitted parameters, and group AC and fitted parameters and association (PDF)
