# Supplementary material for: Viscosities and Densities of Binary and Ternary Mixtures of Aliphatic and Polyaromatic Hydrocarbons: Pyrene +1-Methylnaphthalene + Dodecane at T = (293.15 to 343.15) K. Experiment and Modeling
Source: J Chem Inf Model. 2024 Mar 19;64(8):3192–204. doi: 10.1021/acs.jcim.3c01737 (PMC11040728; doi:10.1021/acs.jcim.3c01737)
Supplement: Supplementary file 1 — ci3c01737_si_001.pdf [file ci3c01737_si_001.pdf]

Supporting Information

Viscosities and densities of binary and ternary mixtures of aliphatic and polyaromatic hydrocarbons: pyrene + 1-methylnaphtalene + dodecane at T = (293.15 to 343.15) K. Experiment and Modeling

Maria José Tenorio[a], Miguel A. Gonzalez[a], Julia D. Magdaleno[a], Inmaculada Suárez[a], Baudilio Coto[a]*

[a]Chemical, Energy and Mechanical Technology Department, ESCET. Universidad Rey Juan Carlos, c/ Tulipán s/n, 28933 Móstoles, Madrid, Spain

---

*To whom correspondence should be addressed

Phone: 34 91 4887072

Fax: 34 91 4887068

E-mail: mariajose.tenorio@urjc.es

Table S1. Standard deviations of all mixtures and all models

	System	Linear	Ratcliff Table 2	Krieger	UNI-Visco Table 8	UNI-Visco* Tables 2 and 8	Guo
Binary mixtures	1% py+mn	0.008	0.023	0.084	0.008	0.015	0.171
	2.5% py+mn	0.062	0.017	0.031	0.065	0.055	0.196
	5% py+mn	0.124	0.040	0.055	0.138	0.118	0.236
	7.5% py+mn	0.194	0.067	0.129	0.221	0.190	0.278
	10% py+mn	0.305	0.090	0.233	0.358	0.313	0.411
	12.5% py+mn	0.359	0.122	0.299	0.424	0.372	0.459
	15% py+mn	0.443	0.151	0.385	0.520	0.457	0.517
Ternary mixtures	1% py r0.1	0.050	0.020	0.053	0.037	0.043	0.054
	2.5% py r0.1	0.047	0.006	0.054	0.008	0.020	0.063
	5% py r0.1	0.039	0.030	0.061	0.066	0.035	0.077
	7.5% py r0.1	0.010	0.026	0.101	0.166	0.120	0.075
	10% py r0.1	0.301	0.258	0.373	0.481	0.427	0.313
	1% py r0.5	0.184	0.049	0.117	0.202	0.206	0.268
	2.5% py r0.5	0.168	0.036	0.101	0.153	0.161	0.261
	5% py r0.5	0.139	0.018	0.074	0.072	0.087	0.250
	7.5% py r0.5	0.087	0.022	0.024	0.039	0.022	0.218
	10% py r0.5	0.095	0.035	0.035	0.084	0.059	0.242
	12.5% py r0.5	0.058	0.042	0.018	0.180	0.151	0.212
	15% py r0.5	0.040	0.058	0.050	0.264	0.232	0.207
	1% py r1	0.203	0.070	0.164	0.234	0.235	0.339
	2.5% py r1	0.206	0.033	0.167	0.203	0.206	0.347
	5% py r1	0.166	0.024	0.128	0.115	0.120	0.319
	7.5% py r1	0.051	0.066	0.027	0.128	0.110	0.100
	10% py r1	0.140	0.059	0.106	0.019	0.010	0.322
	12.5% py r1	0.065	0.030	0.035	0.138	0.130	0.249
	15% py r1	0.040	0.038	0.060	0.270	0.260	0.195
	1% py r5	0.180	0.019	0.219	0.200	0.202	0.301
	2.5% py r5	0.138	0.010	0.176	0.138	0.138	0.266
	5% py r5	0.077	0.008	0.113	0.044	0.047	0.222
	7.5% py r5	0.021	0.023	0.048	0.052	0.047	0.180
	10% py r5	0.063	0.043	0.044	0.143	0.137	0.178
	12.5% py r5	0.071	0.060	0.053	0.177	0.168	0.183
	15% py r5	0.137	0.063	0.111	0.286	0.273	0.196
	1% py r10	0.145	0.054	0.204	0.151	0.154	0.228
	2.5% py r10	0.112	0.052	0.169	0.104	0.107	0.207
	5% py r10	0.052	0.047	0.105	0.024	0.031	0.193
	7.5% py r10	0.029	0.045	0.046	0.072	0.061	0.186
	10% py r10	0.081	0.060	0.050	0.144	0.126	0.186
	12.5% py r10	0.143	0.091	0.108	0.229	0.206	0.229
	15% py r10	0.220	0.220	0.184	0.220	0.220	0.267

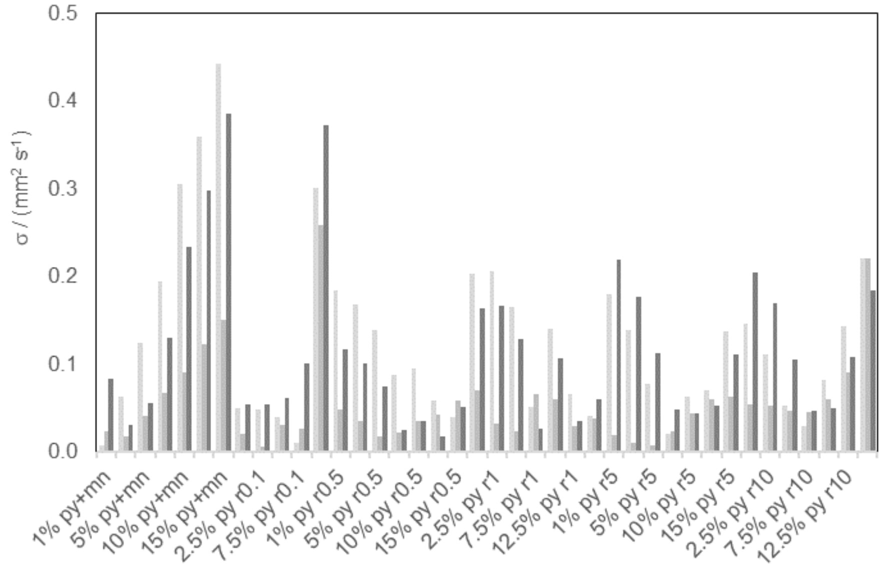

Figure S1. Standard deviations, σ, for all the systems studied using (light grey) the Linear model; (grey) Ratcliff model $\Delta(\ln \nu)_{t3}$; and (dark grey) Krieger model.

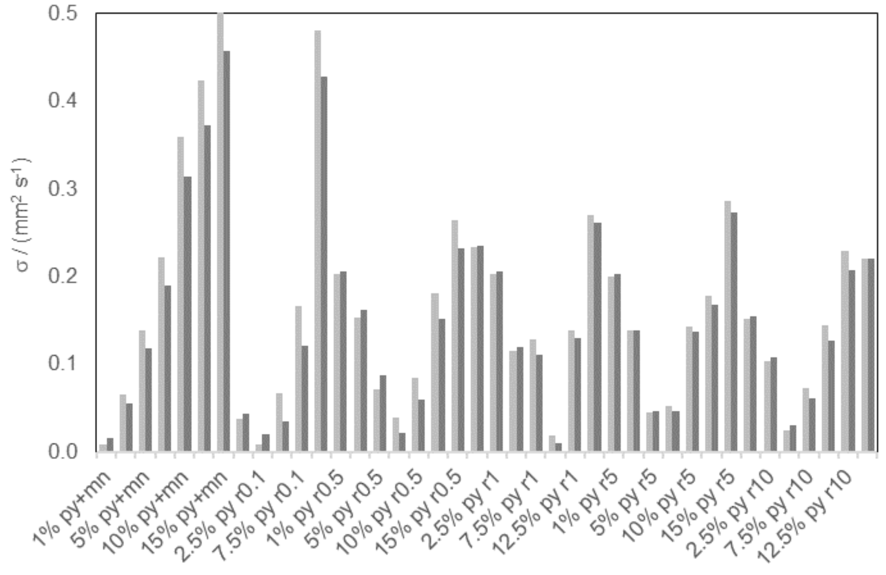

Figure S2. Standard deviations, σ, for all the systems studied using the UNIFAC-Visco model: (grey) group AC and fitted parameters; (dark grey) group AC and fitted parameters and association.
